# High level soluble expression, one-step purification and characterization of HIV-1 p24 protein

**DOI:** 10.1186/1743-422X-8-316

**Published:** 2011-06-22

**Authors:** Baozhong Zhang, Dabin Liu, Zuoyi Bao, Bin Chen, Cun Li, Huanhuan Jiang, Xiaona Wang, Zhiqiang Mi, Xiaoping An, Jun Lu, Yigang Tong

**Affiliations:** 1 State Key Laboratory of Pathogen and Biosecurity, Beijing Institute of Microbiology and Epidemiology, Beijing 100071, China; 2 Beijing YouAn Hospital, Capital Medical University, Beijing 100054, China

## Abstract

**Background:**

P24 protein is the major core protein of HIV virus particle and has been suggested as a specific target for antiviral strategies. Recombinant p24 protein with natural antigenic activity would be useful for various studies, such as diagnostic reagents and multi-component HIV vaccine development. The aim of this study was to express and purify the p24 protein in soluble form in *E.coli*.

**Results:**

According to the sequence of the p24 gene, a pair of primers was designed, and the target sequence of 700 bp was amplified using PCR. The PCR product was cloned into pQE30 vector, generating the recombinant plasmid pQE30-p24. SDS-PAGE analysis showed that the His-tagged recombinant p24 protein was highly expressed in soluble form after induction in *E. coli *strain BL21. The recombinant protein was purified by nickel affinity chromatography and used to react with HIV infected sera. The results showed that the recombinant p24 protein could specifically react with the HIV infected sera. To study the immunogenicity of this soluble recombinant p24 protein, it was used to immunize mice for the preparation of polyclonal antibody. Subsequent ELISA and Western-Blot analysis demonstrated that the p24 protein had proper immunogenicity in inducing mice to produce HIV p24 specific antibodies.

**Conclusion:**

In this work, we report the high level soluble expression of HIV-1 p24 protein in *E. coli*. This soluble recombinant p24 protein specifically react with HIV infected sera and elicit HIV p24 specific antibodies in mice, indicating this soluble recombinant p24 protein could be a promising reagent for HIV diagnosis.

## Background

The human immunodeficiency virus type 1 (HIV-1) is the main cause of the acquired immunodeficiency syndrome (AIDS)[1]. Diagnosis of HIV infection, especially early diagnosis, is one of important part of AIDS prevention and control[2]. Gag protein of HIV-1, a polyprotein of 55 kDa, is one of the most conserved viral proteins. The Gag protein is cleaved by a viral protease to release p17, p24 and p12 during viral maturation[3]. P24 protein is the major core protein of the virus particle and has been suggested as a specific target for antiviral strategies[4]. P24 protein is one of the detecting targets of most diagnostic kits. P24 antigen detection is also helpful for early diagnosis of HIV-infection[5]. The fourth-generation test assays for HIV infection is established on the basis of the p24 antigen detection and is able to find the HIV-infected at an early stage, resulting in shortened diagnostic windows[6]. The p24 protein also can be used as an integral part of any multi-component HIV vaccine[7,8].

A proper recombinant p24 protein with the same antigentic activity as natural p24 protein would be useful for a number of studies. The p24 protein have been produced in a wide variety of systems, including *Escherichia coli*[9]*, Pichia pastoris*[10], plant-based expression system[11,12], baculovirus-insect cell[3], etc. In this study, a recombinant plasmid was constructed to express the His-tagged p24 protein in *Escherichia coli*. The protein was expressed in soluble forms and purified by Ni^2+^-NTA affinity chromatography. Enzyme-linked immunosorbant assay (ELISA) and Western blot analysis demonstrated that the recombinant p24 proteins exhibited good immunoreactivity and immunogenicity.

## Methods

### Strains, plasmids, enzymes and reagents

The *E. coli *strains DH5α and BL21(DE3) were used for cloning experiments and protein expressions, respectively. Both strains were purchased from Invitrogen (Novagen, Shanghai, China). Plasmid pQE30 (Novagen, Darmstadt, Germany) was used for recombinant protein expression. Restriction enzymes, Taq DNA polymerase, and T4 ligase were purchased from TaKaRa Biotechnology Co. (Dalian, China).

### Construction of the plasmid expressing the p24 protein

The HIV-1 p24 open reading frame was amplified from plasmid pHIV which contains the HIV-1 NY5 and LAV strain hybrid genome [13] with the forward primer (5'-GAG GAT CCC CCA TAG TGC AGA ACC TC-3', *BamH*I site underlined), and the reverse primer (CCG GTA CCT TAG AAA ACT CTT GCT TTA TG-3', *Kpn*I site underlined). The PCR product was digested with *BamH*I and *Kpn*I and inserted into the prokaryotic expression pQE30 digested with the same enzymes to create the p24 expression plasmid pQE30-p24.

### Expression of the p24 protein

*E.coli *BL21 transformed with pQE30-p24 was cultured in LB medium supplemented with 50 μg/ml ampicillin for growth at 37°C until the logarithmic phase (at OD600 of 0.5-0.6) and induced by isopropyl-β-D-Thiogalactoside (IPTG) at a final concentration of 1.0 mM for 12 h at 20°C. The bacterial lysates were subjected to 15% SDS-PAGE, and Bandscan5.0 software was applied to assess the expression of the fusion protein.

### Characterization of the solubility of the p24 protein

To assess the solubility of the His-tagged p24 protein, logarithmic phase bacterial cultures were pelleted and suspended in 20 mM Tris-HCl lysis buffer (pH 8.0) supplemented with 100 mM NaCl, 1.0 mM phenylmethyl sulfonylfluoride (PMSF), 50 mg/ml lysozyme and subjected to sonication on ice until clear. The total bacterial proteins were then partitioned into soluble and insoluble fractions by centrifugation at 14,000 × g for 20 min at 4°C. The supernatant (soluble fraction) was collected and the pellets (insoluble fraction), which contained the inclusion bodies, were suspended in deionized water. Both fractions were analyzed in parallel by 15% SDS-PAGE to characterize the solubility of the His-tagged p24 protein.

### Purification of the p24 protein

The supernatant was filtered through a 0.45-μm membrane (Pall Corporation, USA) and then loaded onto a gravity-flow column packed with 2 ml Ni^2+^-NTA resin slurry (Qiagen, Germany). His-tagged p24 fusion proteins were purified following the manufacturer's handbook for high-level expression and purification of 6×His-tagged protein and the yield was quantified using a Coomassie Protein Assay Kit (Biomed, China). 15% SDS-PAGE was performed to validate the identity and evaluate the purity of the target fusion protein.

### Recombinant p24 protein identification

To confirm the presence and the apparent molecular mass of the recombinant proteins expressed in *E. coli*, Western blot was carried out using anti-His antibody (Sigma). The purified recombinant p24 protein were separated by 15% SDS-PAGE, electrotransferred onto a nitrocellulose membrane (GE Healthcare, USA) and blocked with 5% non-fat dry milk in TBS (50 mM Tris-HCl, 150 mM NaCl, pH 7.5) at 37°C for 2 h. After washing 3 times (each 5 min) with TBS containing 0.05% Tween-20 (TBST), the membrane was incubated with horseradish peroxidase-conjugated anti-His monoclonal antibody. Immunoreactive proteins were then visualized using the ECL Western blotting analysis system (Pierce, Rockford, USA).

### Immunoreactivity analysis of the recombinant p24 protein

Human serum samples (n = 90) were obtained from PLA Center for HIV Test, including forty HIV-1 positive samples and fifty HIV-1 negative human serum samples. The protein p24 (1.5 ug/ml in 200 nmol/L NaHCO_3 _pH9.8, 100 ul/well) were coated on ELISA plates (Nunc, Roskilde, Denmark) at 4°C overnight. Plates were then blocked at 37°C for 3 h with 5% non-fat milk and washed four times with PBST. Human sera were added as the primary antibody (1:50 dilution) at 37°C for 1 h. Plates were then washed four times with PBST and incubated with HRP-conjugated goat anti-human IgG (1:3000 dilution) at 37°C for 1 h. Color was developed using TMB solution (Sigma) and absorbance was examined using an ELISA reader at 450 nm.

### Vaccination and HIV-1 p24 specific antibodies detection

Three females BALB/c mice, 8-week-old (purchased from the Center of Experimental Animals, Academy of Military Medical Sciences, Beijing) were injected intradermally on the back and abdomen with 80 μg purified His-tagged p24 protein mixed with complete Freund's adjuvant (100 μl per site). Pre-immune mouse sera were collected prior to immunization. After three immunizations within an interval of 21 days, these immunized mice were sacrificed. Blood samples were collected and stood at 4°C for 2 h, and the sera were aspirated after centrifugation at 4000 rpm for 10 min at 4°C. Serum samples were serially diluted from 1:500, to 1:8192000 and anti-p24 antibodies titer were determined by indirect ELISA with recombinant p24 protein. Western blotting was performed with cultured HIV-1 extracts to detect the antibodies specificity.

## Results

### Construction of p24 prokaryotic expression plasmid

**Figure 1:**
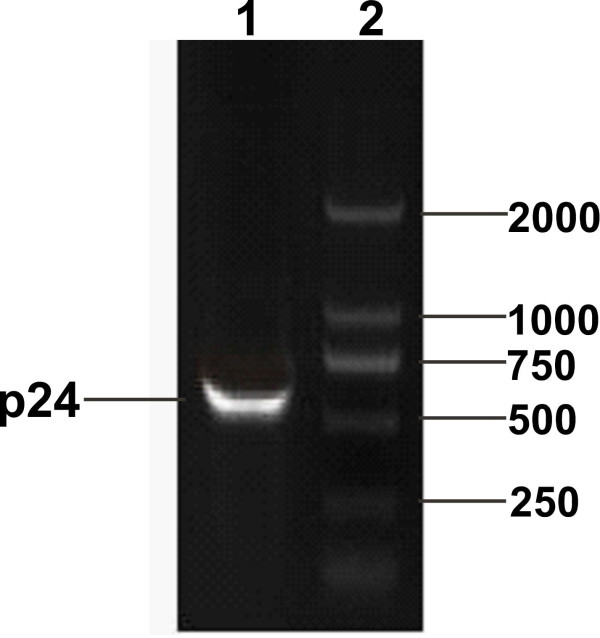
**PCR amplification of HIV p24 gene**. Lane 1, the amplified product of HIV p24 (about 700 bp); Lane 2, DNA marker 2000 (TaKaRa, Dalian, China);

The p24 gene was amplified from plasmid pHIV (Figure 1). The PCR product was digested with *BamH*I and *Kpn*I and inserted into pQE30 digested with the same enzymes to yield the recombinant plasmid pQE30-p24. The pQE30-p24 construct was verified by restriction enzyme digestion and DNA sequencing (data not shown).

### Expression, purification and identification of the recombinant p24 proteins

**Figure 2:**
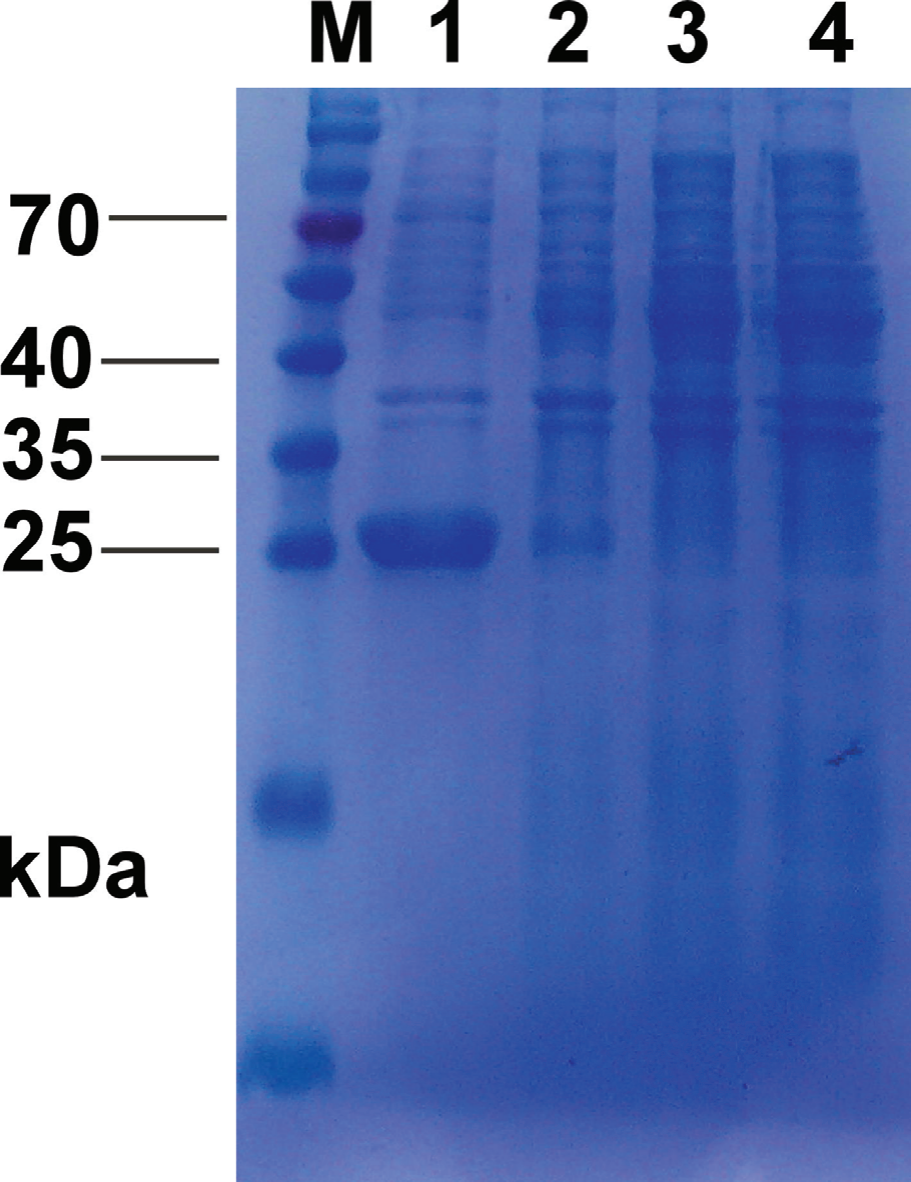
**The SDS-PAGE analysis of recombinant p24 protein**. Lane M, Protein molecular marker; Lane 1, *E.coli *BL21(DE3) whole cell harboring pQE30-p24 after induction with IPTG (about 24 kDa); Lane 2, *E. coli *BL21(DE3) whole cell harboring pQE30-p24 before induction with IPTG; Lane 3, host cell *E. coli *BL21(DE3) (as negative control); Lane 4, *E.coli *whole cell harboring empty plasmid pQE30 after induction with IPTG.

**Figure 3:**
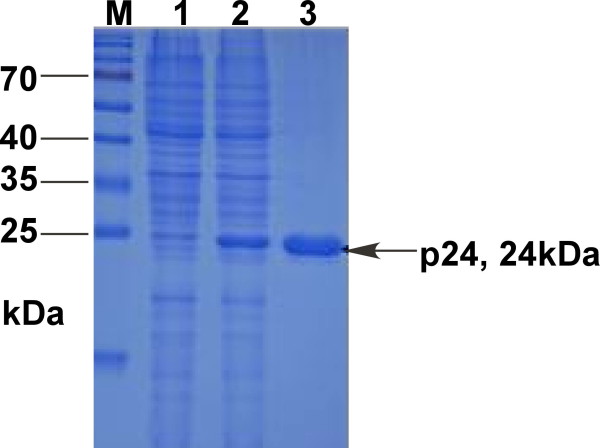
**The SDS-PAGE analysis of the solubility of the recombinant p24 protein**. Lane M, Protein molecular marker; Lane 1, cell extract of *E. coli *harboring pQE30-p24 (pellets); Lane 2, cell extract of *E. coli *harboring pQE30-p24 (supernatant); Lane 3, the recombinant p24 protein purified by the Ni^2+^-NTA agarose gel.

*E.coli *BL21 transformed with pQE30-p24 was cultured in LB medium supplemented with 50 μg/ml ampicillin for growth at 37°C until the logarithmic phase (at OD600 of 0.5-0.6) and induced by isopropyl-β-D-Thiogalactoside (IPTG) at a final concentration of 1.0 mM for 12 h at 20°C (In preliminary experiments, the optimal temperature for obtaining high level expression of p24 was determined to be 20°C). A distinct band of approximately 24 kDa, corresponding to the expected molecular weight of the His-tagged p24 protein, was found only after induction, whereas there was no expression of the p24 protein either in BL21 harboring pQE30-p24 without IPTG induction, BL21 alone or BL21 with pQE30 (Figure 2). Densitometry scan after SDS-PAGE analysis exhibited a relatively high expression level of the recombinant protein, which constituted approximately 20.4% of the total proteins. According to the SDS-PAGE analysis of the soluble fraction and cell debris pellet, the majority of the induced protein was found in the soluble fraction, suggesting that the His-tagged p24 protein was mainly in soluble form (Figure 3).

**Figure 4:**
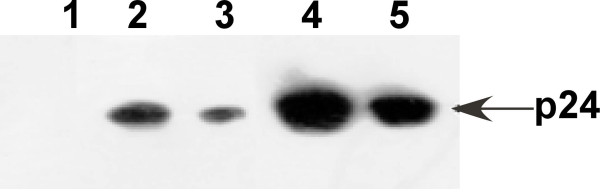
**Western blotting analysis of the recombinant p24 protein**. Lane 1: *E. coli *whole cell harboring empty plasmid pQE30 after induction with IPTG; Lane 2: *E. coli *whole cell harboring pQE30-p24 after induction with IPTG; Lane 3: cell extract of *E. coli *harboring pQE30-p24 after induction with IPTG (pellets); Lane 4: cell extract of *E. coli *harboring pQE30-p24 after induction with IPTG (supernatant); Lane 5: purified His-tagged p24 protein.

Purification of the His-tagged p24 protein was performed with a Ni2+-NTA resin column. The SDS-PAGE analysis of the elution showed a single target band corresponding to the expected molecular weight of p24-HIS (24 kDa) (Figure 3). To confirm the presence of the p24-HIS protein, Western blot was carried out using anti-His antibody. Specific blotting bands were detected at the corresponding positions (Figure 4).

### Immunoreactivity analysis of recombinant p24 protein

In order to evaluate the potential of the recombinant p24 protein as a diagnostic reagent, it was coated on microplates. Indirect ELISA demonstrated that the recombinant p24 protein specifically reacted with HIV-infected human serum samples (positive for all the forty HIV-1 infected human serum samples tested), and did not with most of the normal human sera (for the total 50 uninfected sera tested, only two samples displayed weak positivity, which may due to the cross reactivity of residual bacterial components) (Table 1). These results suggest this recombinant protein possess proper immunoreactivity.

**Table 1:** Efficacy of the recombinant p24 for detecting antibodies in HIV-infected individuals.

HIV infection	p24 ELISA result		Total
	**+**	-	
Positive sera	40	0	40
Negative sera	2	48	50

Total	42	48	

P < 0.001 (χ^2 ^test)

Microtitration plate wells were coated with 150 ng recombinant p24 per well. Sera were used at 1:50 dilution. HRP-conjugated goat anti-human IgG was used at 1:3000 dilution.

### The immunogenicity of p24 protein expressed in *E.coli*

**Figure 5:**
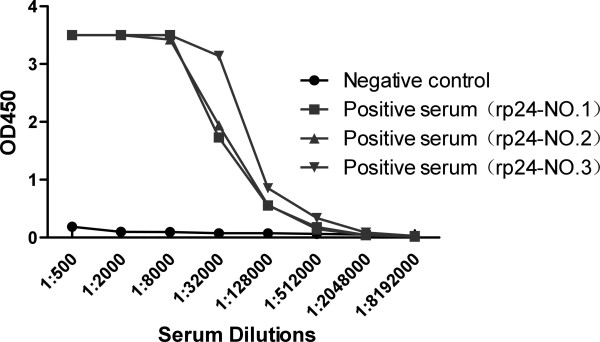
**Titer of anti-p24 antibodies in the immunized mouse sera determined by indirect ELISA**. The prei-mmune serum served as the negative control. Serum samples were diluted at 1/500, 1/2000, 1/8000, 1:32000, 1:128000, 1:512000, 1:2048000, 1:8192000.

Indirect ELISA and Western blot were performed to access the immunogenicity of the recombinant protein. After the mice received three times of immunization at 21 days intervals, these immunized mice were sacrificed and the sera were isolated. For the indirect ELISA, serum samples were diluted at 1/500, 1/2000, 1/8000, 1:32000, 1:128000, 1:512000, 1:2048000, 1:8192000, respectively, and were used to react with the recombinant p24 proteins, with pre-immune serum served as the negative control. The titer of immunized mice serum was determined to be more than 1:128000 by indirect ELISA (Figure 5).

**Figure 6:**
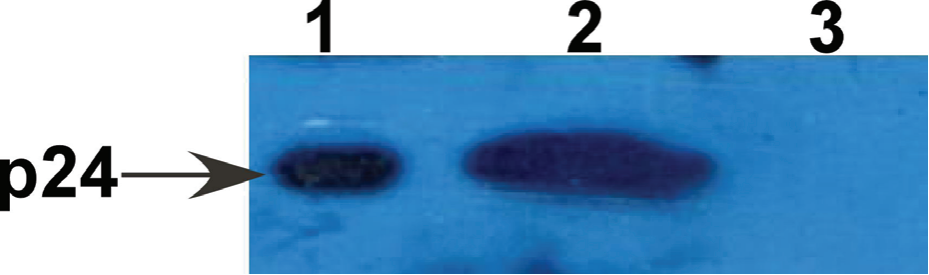
**Antibody specificity of mouse sera immunized with the recombinant p24 protein**. Lane 1: recombinant p24 protein. Lane 2: cultured HIV-1 extract. Lane 3: BSA (negative control). Arrow at the left indicates the position of p24 protein.

In the Western blot analysis of cultured HIV-1 extract, the sera from immunized mice were used as the capture antibody and HRP-labeled goat anti-mouse immunoglobulin G were used as the secondary antibody. Recombinant p24 protein was used as the positive control and bovine serum albumin (BSA) as negative control. Western blotting results showed that the immunized mouse serum could react with cultured HIV-1 extract and recombinant p24 protein (Figure 6), while it could not react with BSA (Figure 6). These results indicated that p24 protein expressed in E.coli had proper immunogenicity and could induce mice to produce specific antibodies against HIV-1.

## Discussion

The bacterial expression system is the most universally used, as it is relatively inexpensive, and with ease of manipulation and a rapid growth rate[14]. Problematically, with the bacterial expression system, inclusion bodies are often produced due to incorrect folding and thus require denaturation and renaturation before further use. These processes may complicate or inhibit recombinant protein production. Use of a low culture temperature, low inducer concentrations and the co-expression of molecular chaperones can promote the soluble expression of recombinant proteins [15]. In this study, the soluble p24 protein was highly expressed at a low temperature (20°C) and a low IPTG concentration (0.6 mM). To further increase the recombinant protein solubility, we chose *E. coli *BL21 (DE3) as the host strain, which was previously demonstrated to be appropriate for soluble expression[15]. By adopting these strategies, the recombinant p24 protein was expressed in soluble form and was easily purified.

It is reported that protein primary structure may influence soluble protein expression. Change of the amino acids in proteins (especially cysteine) may significantly influence the folding and alter the conformation of the protein. Studies conducted by Strandberg[16] and Wetzel et al[17] have shown that a few amino acid changes can remarkably alter the soluble expression of target protein, and Rinas et al[18] have also reported that individual amino acid changes involving cysteine drastically changed the solubility of recombinant proteins.

**Figure 7:**
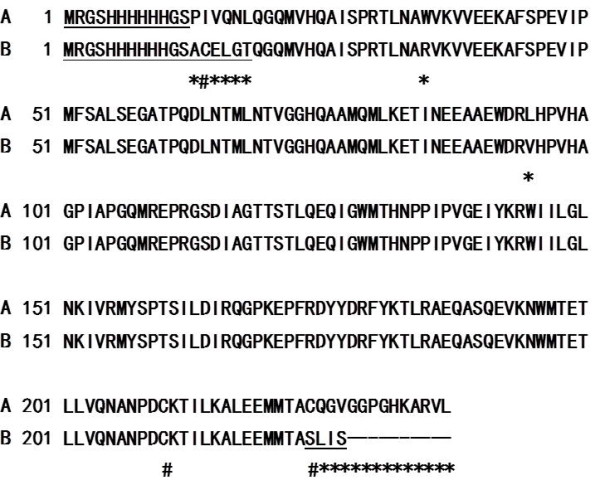
**Sequence comparison between soluble and insoluble p24 protein synthesized in *E. coli***. A. Sequence of the soluble p24 protein in this report; B. Sequence of the insoluble p24 protein reported by Gupta. * indicates the amino acid difference. # indicates the Cys residue. The artificial regions are underlined.

Up to now, there are a few reports about of HIV p24 protein expression[10,12,19,20]. Some studies have used complicated strategies to achieve soluble p24 expression[7,21,22]. For our knowledge, up to now, no one has reported a simple method for soluble p24 protein expression. Like this report, Gupta et al[9] used the pQE30 to express recombinant p24 protein, but the recombinant p24 protein was produced as inclusion bodies. Our soluble p24 protein differed from Gupta's insoluble p24 protein in a few amino acids, including cysteine residues. The solubility of our p24 protein may be attributed to Cys230 which located in the C-terminal (Figure 7). The Cys230 and Cys210 are likely to form an intra-chain disulfide bonds, resulting in a small ring. This structure may promote the proper fold of the recombinant protein.

The early diagnosis of HIV infection is very important for prevention of HIV spread and ensuring safety of blood products[19]. Currently most diagnostic reagents are based on the method of ELISA for detection of antibodies against HIV proteins[19,23]. Synthetic peptides and/or recombinant proteins spanning the envelope (gp41 of HIV-1 and gp36 of HIV-2 respectively) and the core (p24) proteins are often used as capture antigens[19]. Antibodies directed against p24 appear early in HIV infection and are reported to decline with progression of the disease due to increasing antigenemia[24]. It is reported that synthetic peptides (9-53-mer) corresponding to p24 did not give satisfactory results[9]. Recombinant p24 protein may be a better option compared with synthetic peptides. In this paper, we report the high level soluble expression of HIV-1 p24 protein in *E. coli*. This soluble recombinant p24 protein specifically reacts with HIV-1 infected sera. To study the immunogenicity of this soluble recombinant p24 protein, it was used to immunize mice for preparation of polyclonal antibody. Subsequent ELISA and Western-Blot analysis demonstrated that the p24 protein had proper immunogenicity in inducing mice to produce HIV-1 p24 specific antibodies. Recently the fourth generation HIV assays include the p24 antigen detection, thus high affinity antibodies with high specificity for p24 is a pre-requisite. A good recombinant protein may be a great help to manufacturing qualified p24 antibodies. Our soluble p24 protein which has exhibited good immunoreactivity and proper immunogenicity may help us to gain high affinity monoclonal antibody in the further research.

## Conclusions

In this study, we have highly expressed soluble recombinant HIV-1 p24 protein in *E.coli*. This soluble p24 had good immunoreactivity and immunogenicity. Its characteristics suggest that this recombinant p24 protein holds promise for assembling the HIV diagnostic kits, as well as for the development of the fourth generation HIV test kits.

## Competing interests

The authors declare that they have no competing interests.

## Authors' contributions

BZ and DL carried out most of the experiments and drafted the manuscript. YT and JL have critically revised the manuscript and made the experimental design. ZB, BC, CL, HJ, ZM and XA helped in experiments. All authors have read and approved the final manuscript.
